# Surgical Aphorisms

**Published:** 1887-04

**Authors:** J. J. Berry

**Affiliations:** Portsmouth, N. H.


### SELECTIONS.

### Surgical Aphorisms.

BY J. J. BERRY, M.D., OF PORTSMOUTH, N. H.

Surgical literature does not always supply the information most essential to the operator, neither is the skill or dexterity of the surgeon a guarantee of successful results.

To the minor details of surgical care, therefore, upon which I believe hinges the great progress and successes of the day, I desire to call attention. If the views expressed should savor of dogmatism, they may be pardoned as being the result only of well settled convictions.

Do not operate upon patients who are under novel conditions of locality, temperature, domestic life, etc. Sudden and wellmarked variations of temperature and humidity have their definite and peculiar effects upon the course and termination of operative cases.

The secretions should be fully regulated prior to every operation, and should form an important portion of the after-treatment.

The administration of quinine seems to us to have a decided effect upon the intensity of surgical fever.

By chronic alcoholism shock is rendered more profound and persistent, suppuration more liable, secondary hæmorrhage more imminent, and internal complications more frequent.

The habitual use of opium has a very unfavorable effect upon the mortality of surgical cases.

To the method of Lister are we indebted for two things, namely, aseptic conditions and attention to details. In some other particulars it is objectionable and cannot be considered as fully abreast of the times. In the large majority of cases the use of strong antiseptic solutions is reprehensible, the latter are decidedly irritating, and by their action upon the tissues, exert an unfavorable influence upon the blood supply of a given part. The good effects attributed to them may in reality be due to irrigation rather than to germicidal properties. The free use of water, rendered sterile by previous boiling, will be found to meet most indications. We are persuaded that ordinarily there is no suppuration in deep wounds which are aseptic and under proper care, and moreover that it is only under certain conditions that germs are harmful.

It may be stated that in many cases drainage tubes of whatever variety are of doubtful utility. Those of small calibre are manifestly injurious, large sizes are a source of irritation, and delay healing in deeper portions of the wound. Although certain cases seem now to require them, recent advances indicate that in many varieties of wounds we shall soon dispense with all methods for securing drainage, in fact, there will be nothing to require it.

Immediate union is prevented and delayed by certain conditions of the tissues. The common practice of dissecting out tumors and the like, with the handle of the scalpel, should be pursued only when absolutely necessary. The result of such a procedure is disastrous to the vitality of the parts; minute blood-vessels are torn through and off, nerve filaments are bruised, and minute portions of tissue are deprived of their blood supply and become necrotic. It is far better to include portions of healthy tissue in an exsected mass, than to economize in this particular and produce a lacerated wound. The same thing results when considerable portions of tissue are caught in the teeth of artery forceps and twisted, or when a cautery iron is applied to a bleeding surface, or when strong styptics are applied to control hæmorrhage.

For similar reasons, a wound should not be closed until the slightest oozing from its surface has been arrested. Not only does coagulated blood prevent coaptation, but unless rapidly absorbed is sure to invite suppuration. Every wound should therefore be packed with some aseptic absorbent material before being permanently closed. In fine, the opposing surface should be as free as possible from all sources of irritation of whatever nature.

Histology teaches that adipose tissue possesses a very low degree of vitality, and this fact is borne out by clinical experience. It is highly important therefore, that every wound should be freed of every accessible portion of a substance so inimical to repair.

As a rule no operation is well done unless rapidly performed. The exposure of a wound for a long time to the atmosphere (contaminated or not) together with frequent and rough manipulation has no good effect upon its subsequent behavior.

Heavy and impermeable dressings possesses no advantage over more agreeable ones. Perfect protection from the air can be equally well secured by sealing the wound with some mild protective application.

The irrigation of abscess cavities with antiseptic solutions has usually no other than a deodorizing effect upon the same. Hot water has often an equally good action upon suppurating surfaces.

Perfect rest is one of the essentials of good treatment. All movement of injured parts is to be avoided, and muscular action in the vicinity of wounds should be carefully prevented by splints, adhesive plaster, and dressings, which can be readily applied and as easily removed; one of the good points of the Lister or Gamgee dressing is that the wound is allowed to remain a long time undisturbed.

Physical exertion, even when moderate in degree, delays the processes of repair; other things being equal, minor wounds heal more rapidly if the patient be confined to the bed.

At the present time we are not justified in claiming that one given method of wound dressing is superior to all others and offers uniformly successful results. Simplicity in methods of operating and surgical treatment has become an important desideratum, but to a great degree is success dependent upon the first dressing of a wound.

